# Torsion of an Epididymal Cyst: A Rare Finding on Scrotal Exploration for Acute Scrotum

**DOI:** 10.1155/2020/8858606

**Published:** 2020-11-22

**Authors:** A. M. Omar Mohamed Ozaal, B. Pragalathan, S. Lavanya, S. T. Sarma

**Affiliations:** ^1^General Surgery, University Surgical Unit, Teaching Hospital Jaffna, Sri Lanka; ^2^University Surgical Unit, Teaching Hospital Jaffna, Sri Lanka

## Abstract

Torsion of epididymal cyst (EC) is an exceedingly rare cause of acute scrotum in both children and adults. We add our case as the ninth case to literature which was an 8-year-old child presented with features of acute scrotum with history of EC on conservative management. Doppler sonography showed perfused normal bilateral testes and a 4.1 × 1.7  cm septate cystic lesion of right epididymis. On scrotal exploration, we found a haemorrhagic cystic lesion attached to the upper pole of right testis and twisted for 540 degrees with normal testis and appendage. Cyst was excised, and histopathology revealed a haemorrhagic EC. Our case was peculiar due to, presenting as acute scrotum in a child of 1-10 years age group who was conservatively managed for right-sided EC and presence of 540 degrees torsion.

## 1. Introduction

Acute scrotum can be considered as a urological equivalent of acute abdomen in general surgery. There are myriad of causes for acute scrotum ranging from torsion of testes or appendages, traumatic injuries, infections and inflammatory conditions of testes. Several conditions will require scrotal exploration, and patients should be assessed clinically along with imaging studies as it is time sensitive.

Epididymal cyst (EC) is a benign lesion in children and ranges from 5% to 20% in the literature in contrast to adults where it is common [1]. They are of unknown aetiology, common in testicular maldescent, and managed conservatively with spontaneous regression in most instances [2]. Torsion of EC is rare and a scarce finding on scrotal exploration for acute scrotum. There were eight paediatric cases reported in the literature (Table 1), and among them, seven cases were all adolescents, and one was an infant [1, 3–5]. Our patient is an 8-year-old child and the first case of EC torsion in this age group to be reported and added as the ninth case to the literature.

## 2. Case Presentation

An 8-year-old boy was admitted to our casualty ward with persistent scrotal pain of 2 days duration associated with nausea and one episode of vomiting. He was treated by local general practitioner with analgesics the day prior and denied preceding trauma to scrotum. But he was investigated for right hemi-scrotal pain without any history of trauma 2 months back in a peripheral hospital. He was diagnosed to have a right-sided EC and opted for conservative management.

**Figure 1:**
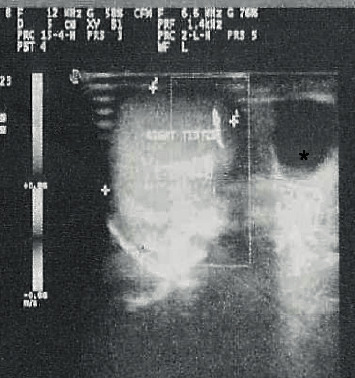
Ultrasound image of right testis with epididymal cyst^∗^ (on acute presentation).

On examination, right hemi-scrotal tenderness and high riding right testis were found. Left hemi-scrotal palpation, abdominal examination, and hernial orifices were unremarkable. Urgent sonography with Doppler showed a septate cystic lesion of right epididymis, sized 4.1  cm × 1.7  cm (Figure 1) along with bilateral normally echogenic and perfused testes.

**Figure 2:**
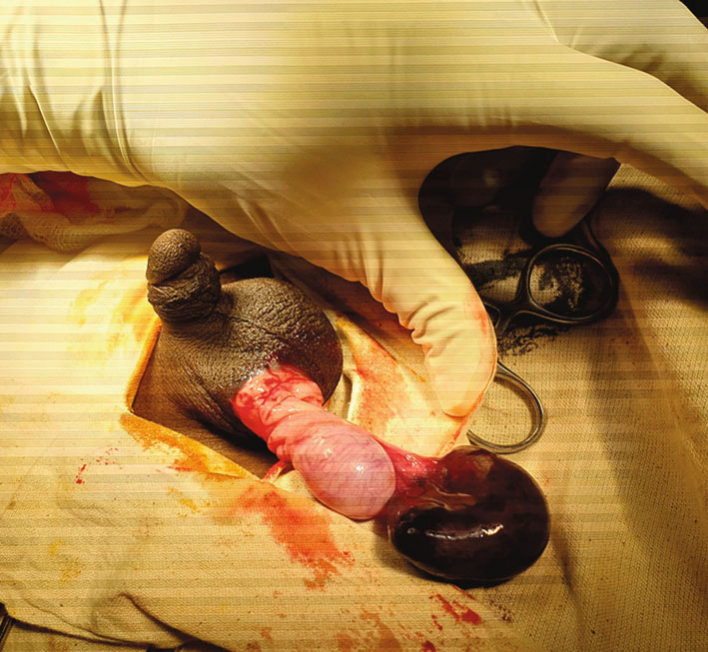
Twisted right Epididymal cyst with normal testis.

Scrotal exploration under general anaesthesia was performed which revealed oedematous tunica vaginalis, age appropriately sized right testis, normal testicular appendage, and 4  cm × 2  cm sized dark haemorrhagic cystic lesion. The lesion appeared to be attached to the upper pole of right testis and was twisted for 540 degrees (Figure 2).

**Figure 3:**
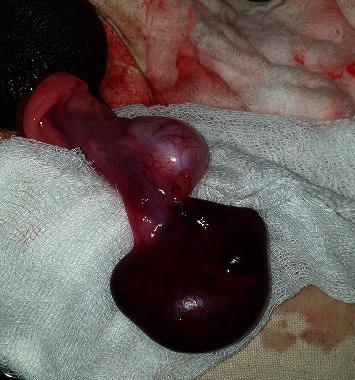
Untwisted hemorrhagic right Epididymal cyst.

**Figure 4:**
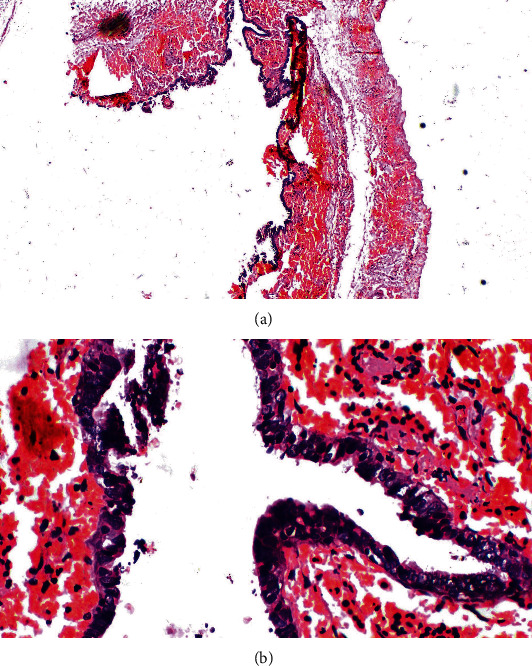
(a) Low and (b) high power views of the epididymal cyst wall with columnar epithelium showing congestion and haemorrhage.

Cyst was untwisted (Figure 3) and excised, and the testis was fixed. Histopathological assessment revealed haemorrhagic EC (Figure 4). Patient was discharged the next day following unremarkable recovery, with analgesics and oral antibiotics.

## 3. Discussion

Epididymal cysts occur in 94.9% of boys older than 10 years, and 71.2% of them are older than 14 years [2]. In our case, the patient was 8 years old and presented with features of an acute scrotum. At the time of admission to ward, neither history of previous ultrasound evidence of a right-sided EC was provided by the patient and guardian nor the previous medical records were available with the patient.

Our line of management was towards torsion of testis derived from the acuteness of presentation with swollen and high ridden testis. Even though Doppler sonography showed a perfused right testis, the patient had persistent scrotal pain with few bouts of vomiting. This prompted us to go ahead with scrotal exploration. Torsion of an EC was not considered into the differential diagnosis due to its rarity over a torsion of testicular appendage.

Intraoperative finding of a twisted haemorrhagic cystic lesion arising from the epididymal location of the right testis attached to its upper pole, confirmed the diagnosis of a torsed EC and led us to carry out excision. The twist was 540 degrees, whereas in the literature, it ranged from 360 to 720 degrees [1, 3–5]. In line with majority of the literature, there was no associated prior scrotal trauma.

Particularity of our case was due to being the first case of torsed EC in a child of 1-10 years age group and being torsed for 540 degrees. It is the second case of a right-sided EC and has an acute presentation in previously diagnosed case of an EC. Bleve et al. [3] reported the first case of torsed right sided EC and the remaining seven cases in the literature (Table 1) are left sided lesions.

Erikci et al. [1] also have reported a case of EC torsion in an 11-year-old boy who was put on conservative management for an EC [1]. Presentation with features of acute scrotum such as pain and swelling was similar among all reported cases [1, 3–5]. Management of an EC is usually conservative as 60% of the cases regress on its own more importantly if less than 3 cm in size [2].

## 4. Learning Points

In conclusion, in line with literature, it is acceptable to perform elective surgery in children for ECs more than 10 mm in size, which are symptomatic, and which are persistent in nature [2].

In cases of ECs opted for conservative management, patients and guardians should be advised on symptoms of acute scrotum and when to seek immediate medical help.

We emphasize the importance of adequate background history taking from the patient as well as the guardian. And it can help to consider a torsed EC as a differential diagnosis in acute scrotum, even though it is rare but important in cases with past history of an EC.

## Figures and Tables

**Table 1:** List of cases in literature with brief description.

Case no.	Authors	Brief description
1^st^ case	RI. Kaye et al., 1990	Left-sided 360° torsed EC in a 13-year-old boy
2^nd^ case	N. Liolios et al., 1997	Left-sided 360° torsed EC in a 6-month-old male
3^rd^ case	E. Yılmaz et al., 2004	Left-sided 720° torsed EC in a 13-year-old boy with history of scrotal trauma
4^th^ case	V. Erikçi et al., 2013	Left-sided 720° torsed EC in an 11-year-old boy on conservative management for EC with no history of scrotal trauma
5^th^ case	Y. Akın et al., 2014	Bilateral simple ECs in a 14-year-old boy with no history of scrotal trauma
6^th^ case	M. Ameli et al., 2015	Left-sided 720° torsed EC in a 14-year-old boy with history of scrotal trauma
7^th^ case	C. Bleve et al., 2018	Right-sided 720° torsed EC in a 16-year-old boy with no history of scrotal trauma
8^th^ case	M. Messina et al., 2019	Left-sided twisted EC in a 13-year-old boy with no history of scrotal trauma
This case		Right-sided 540° torsed EC in an 8-year-old child with no history of scrotal trauma who was on conservative management for EC.
